# Beyond intervention: The clinical meaning of non-intervention in serious illness

**DOI:** 10.1017/S1478951526102545

**Published:** 2026-05-04

**Authors:** João Carlos Geber-Júnior

**Affiliations:** 1 Center for Oncologic Intercurrence Care (CAIO), Cancer Institute of the State of São Paulo (ICESP), São Paulo, Brazil; 2 Palliative Care Support Team, Hospital Samaritano Higienópolis, São Paulo, Brazil

## Introduction

Modern medicine has been profoundly shaped by its capacity to intervene. Advances in biomedical science have transformed the diagnosis and treatment of disease, enabling clinicians to alter biological processes that once appeared irreversible. The development of antimicrobial therapy, organ transplantation, advanced oncologic treatments, and intensive care technologies has expanded the boundaries of what medicine can accomplish. Within this landscape, clinical competence is frequently defined through the ability to act: to diagnose accurately, intervene effectively, and modify the trajectory of illness.

Yet serious illness repeatedly exposes the limits of this model. Progressive disease, advanced frailty, and the proximity of death confront clinicians with situations in which the underlying condition cannot be reversed. In these contexts, the traditional framework of medicine – organized around intervention – begins to falter. The central question is no longer simply what medicine can do, but what care means when intervention is no longer capable of resolving the patient’s condition.

Modern medicine is defined by its capacity to intervene, yet the care of serious illness repeatedly confronts clinicians with experiences that cannot be repaired.

These moments reveal a structural tension within contemporary medical culture. Medicine has developed extraordinary tools for modifying disease, but far fewer conceptual tools for understanding how care should unfold when modification is no longer possible. Situations of therapeutic limitation are, therefore, sometimes experienced as moments in which medicine itself reaches an endpoint.

However, this interpretation may be misleading. The limits of intervention do not necessarily represent the limits of care. Instead, they reveal dimensions of medicine that become visible precisely when biomedical action is no longer sufficient.

## The limits of intervention

The success of modern medicine has reinforced the assumption that illness should be approached primarily as a problem to be corrected. Clinical training emphasizes diagnostic precision, procedural competence, and the application of therapies designed to restore physiological stability. This orientation has produced remarkable progress in many fields of medicine.

Yet the same framework becomes strained when clinicians encounter forms of suffering that cannot be repaired through biomedical modification. Progressive neurological disease, advanced cancer, and complex multimorbidity often unfold despite technically appropriate treatment. In these circumstances, intervention may gradually lose its capacity to meaningfully alter the underlying trajectory of illness.

The difficulty lies not only in the biological limits of treatment but also in the conceptual structure through which medicine interprets illness. When care is defined primarily through intervention, situations in which intervention becomes ineffective may appear as therapeutic voids.

However, illness disrupts far more than biological systems. As Cassell argued, suffering arises when illness threatens the integrity of the person rather than the functioning of the body alone (Cassell 1982). Serious illness alters identity, relationships, and expectations for the future. Anthropological perspectives similarly emphasize that illness unfolds within narratives through which individuals interpret their experience (Kleinman 1988).

From this perspective, the clinical encounter cannot be reduced to the correction of pathology alone. It also involves engaging with the human meanings that illness generates. Such meanings often extend beyond biomedical classification and have been described as forms of suffering that resist purely clinical framing (Milan-Youssef and Geber-Junior 2026).

## Therapeutic non-intervention

Within this broader framework, the concept of **therapeutic non-intervention** becomes essential.

Therapeutic non-intervention does not refer simply to the absence of treatment. Rather, it describes a deliberate clinical recognition that certain dimensions of illness cannot be resolved through biomedical modification and, therefore, require forms of care that extend beyond intervention.

In many clinical contexts, the decision not to intervene is interpreted as therapeutic limitation or withdrawal. Yet such interpretations overlook the possibility that non-intervention itself can represent a meaningful form of care.

Therapeutic non-intervention performs **3 clinical functions** in the care of patients with serious illness.

First, it involves **recognition**. Clinicians acknowledge that some forms of suffering cannot be repaired through biomedical intervention. Illness may disrupt identity, meaning, and personal narratives in ways that no treatment can fully restore.

Second, it requires **discernment**. Physicians must evaluate when additional interventions are unlikely to provide meaningful benefit and may instead impose burdens that outweigh their potential advantages.

Third, therapeutic non-intervention entails **accompaniment**. Even when curative treatment is no longer possible, the clinician’s responsibility to remain engaged with the patient does not disappear. Care continues through attention to suffering, communication, and relational presence.

Through these functions, non-intervention becomes not a withdrawal of medicine but a transformation of its goals.

Non-intervention is, therefore, not the absence of medicine; it is the moment when medicine recognizes the limits of its own tools.

## What non-intervention reveals about medicine?

Therapeutic non-intervention illuminates a deeper dimension of clinical practice. When intervention is no longer capable of altering the disease trajectory, the relational and interpretive aspects of medicine become more visible. These dimensions have been explored in relation to the temporal expirience of prognosis and the evolving presence of the clinician in serious illness (Geber-Junior and Forte 2025).

Clinicians accompany patients through conversations about prognosis, uncertainty, and the meaning of illness. They help patients and families navigate decisions about treatment preferences, goals of care, and the balance between prolonging life and preserving comfort.

These encounters require attentiveness, ethical reflection, and the capacity to tolerate uncertainty. Such capacities are rarely emphasized within models of medicine centered primarily on technical intervention. Yet they represent essential dimensions of professional competence.

Palliative care has long recognized that the relief of suffering and the preservation of dignity remain central goals of medicine even when disease cannot be reversed (Chochinov 2002). Within this context, clinicians frequently engage with forms of distress that are existential, relational, or narrative in nature rather than purely biological (Breitbart et al. 2015).

Indeed, it is often at the limits of intervention that the ethical depth of medicine becomes most visible.

## Beyond intervention

Recognizing the clinical meaning of non-intervention does not diminish the achievements of modern medicine. The capacity to diagnose and treat disease remains one of the most powerful tools for alleviating human suffering. Rather, the concept of therapeutic non-intervention expands our understanding of care by acknowledging that the goals of medicine may change when cure is no longer possible.

When intervention is beneficial, it should be pursued with rigor and skill. Yet when intervention reaches its limits, clinicians must also recognize the forms of care that become possible beyond those limits.

Therapeutic non-intervention reminds us that medicine does not end where intervention ends.

## Conclusion

Serious illness exposes a fundamental truth about medicine: not all suffering can be resolved through biomedical intervention. When clinicians encounter these limits, they face the challenge of redefining what care means.

Therapeutic non-intervention offers a framework for understanding these moments. Rather than representing abandonment or failure, non-intervention reflects the recognition that medicine’s responsibilities extend beyond the modification of disease.

In this sense, the limits of intervention do not diminish the scope of medicine. They reveal it. They also suggest that suffering may assume a formative role within clinical practice, shaping how physicians come to understand care itself (Geber-Junior 2025).
